# Optimized approach for Ion Proton RNA sequencing reveals details of RNA splicing and editing features of the transcriptome

**DOI:** 10.1371/journal.pone.0176675

**Published:** 2017-05-01

**Authors:** Roger B. Brown, Nathaniel J. Madrid, Hideaki Suzuki, Scott A. Ness

**Affiliations:** 1 Department of Internal Medicine, Division of Molecular Medicine, University of New Mexico Health Sciences Center, Albuquerque, New Mexico, United States of America; 2 UNM Comprehensive Cancer Center, University of New Mexico, Albuquerque, New Mexico, United States of America; Xiamen University, CHINA

## Abstract

RNA-sequencing (RNA-seq) has become the standard method for unbiased analysis of gene expression but also provides access to more complex transcriptome features, including alternative RNA splicing, RNA editing, and even detection of fusion transcripts formed through chromosomal translocations. However, differences in library methods can adversely affect the ability to recover these different types of transcriptome data. For example, some methods have bias for one end of transcripts or rely on low-efficiency steps that limit the complexity of the resulting library, making detection of rare transcripts less likely. We tested several commonly used methods of RNA-seq library preparation and found vast differences in the detection of advanced transcriptome features, such as alternatively spliced isoforms and RNA editing sites. By comparing several different protocols available for the Ion Proton sequencer and by utilizing detailed bioinformatics analysis tools, we were able to develop an optimized random primer based RNA-seq technique that is reliable at uncovering rare transcript isoforms and RNA editing features, as well as fusion reads from oncogenic chromosome rearrangements. The combination of optimized libraries and rapid Ion Proton sequencing provides a powerful platform for the transcriptome analysis of research and clinical samples.

## Introduction

High-throughput next-generation sequencing has become a standard method to study the complexities of the genome and transcriptome. In particular, RNA-sequencing has the ability to provide multiple types of information, including gene expression levels, alternative RNA splicing, sequence variants such as inherited single nucleotide polymorphisms (SNPs), and post-transcriptional variants introduced by RNA editing as well as gene fusions resulting from chromosomal translocations. For example, recent studies have demonstrated the importance of changes in RNA editing [1] and alternative RNA splicing in cancer [2, 3], focusing interest on the detection of these events in clinical samples. In practice however, routine methods of RNA-Sequencing can produce results that fail to adequately detect all the complexities of the transcriptome. Methods that rely on oligo-dT primed cDNA synthesis may produce 3’-end bias, and random priming methods may introduce sequence specificities [4].

Genome-wide assays have estimated that as many as 94% of human genes produce multiple RNA isoforms through alternative RNA splicing [5]. However, many of the isoforms are expressed at low levels, and their importance is debated [6]. For example, the human MYB gene, which encodes a transcription factor capable of regulating differentiation and proliferation, can produce as many as 60 different RNA isoforms, which encode at least 20 different versions of the Myb protein [7, 8]. All of the Myb variants include the same N-terminal DNA binding domain but are predicted to have different transcriptional activities since changes in the C-terminal domains alter the stability and transcriptional specificity of the Myb protein [9–13]. So changes in alternative RNA splicing observed in leukemias and tumors [7, 8] could result in the production of Myb variants with altered, perhaps oncogenic activities [14, 15]. Complete analysis of the isoforms encoded by MYB and similar genes requires both adequate detection of the isoforms that are expressed and the ability to predict which protein variants will be encoded [14, 15].

To be effective, measurements of alternative RNA splicing require high sensitivity to detect rare isoforms and even coverage across the entire lengths of transcripts so that all exons are measured accurately. Specialized library preparation methods have been developed to detect rare transcripts or to analyze individual human cells [16]. However, there are several methods that are used for most RNA-seq experiments, and each approach has its own drawbacks. Priming from the 3’-polyA tail enriches for protein-coding transcripts, but can lead to 3’-end bias. Digestion or fragmentation of the RNA followed by ligation of the RNA fragments to directional primers captures both protein-coding and non-coding RNAs but depends on relatively inefficient RNA ligation steps. Random priming for cDNA synthesis can introduce sequence bias. In addition to library methods, there are also differences in next-generation sequencing platforms: the largest instruments provide cost efficiencies, but the large capacities and high cost of reagents make them impractical for use in small laboratories, when only a few samples need to be analyzed or when rapid turnaround times are required. In contrast, the Ion Proton/S5 platform can produce RNA-Sequencing results for a small number of samples in as little as 24 hours, but the technology is less well developed and most data analysis tools are optimized for data produced on larger platforms [17]. We explored whether the Ion Proton/S5 platform could be utilized for rapid, small-scale RNA-seq assays that would still provide the advanced features of transcriptome analysis, such as identification of isoforms formed through alternative RNA splicing or variants introduced through RNA editing. We tested a variety of RNA-seq library methods to determine the advantages and disadvantages of each for more advanced transcriptome analyses. We found significant differences in the results when different methods of RNA-seq library preparation were compared. The results have important implications for the analysis of advance transcriptome features, such as alternative RNA splicing.

## Materials and methods

### Cell culture and RNA extraction

Jurkat T cells (ATCC, TIB-152), a human T lymphoblastoid cell line derived from an acute T cell leukemia, were cultured in RPMI-1640 Medium (SIGMA, R8758) supplemented with 10% fetal bovine serum (SIGMA, F4135), Trypsin-EDTA, RPMI, phosphate buffered saline, NewBorn calf serum and antibiotics (A/A) (SIGMA, A5959). Cultures were maintained at 37°C, and split 2–3 times per week.

Cytokine-mobilized CD34+ cells (purchased from the Fred Hutchison Cancer Research Center Large-Scale Cell Processing Core) were cultured in StemSpan™ SFEM II media (StemCell, 09605) and supplemented with StemSpan™ CC110 expansion supplement (StemCell, 02691) for CD34+ cells. Jurkat and CD34+ cells were collected 24 hours after plating.

Cell counting was conducted with a glass hemocytometer and coverslip. Trypan Blue-treated cell suspension was used to apply to the hemocytometer to exclude live cells from staining. Total RNA was isolated from cells using RNeasy® Mini Kit (Qiagen, # 74104) according to manufacturer’s instructions. An on-column DNase digestion step for further residual DNA removal was included. Extracted total RNA was transferred to −80°C and stored there until sample processing.

### Random priming method

The Ribogone kit (TaKaRa Clontech, 634847) was used to deplete ribosomal and mitochondrial RNAs. The RiboGone exclusively targets the depletion of 5S, 5.8S, 18S, and 28S nuclear rRNA sequences as well as 12S mtRNA sequences on the basis of hybridization and RNase H digestion. The Clontech SMARTer Universal Low Input RNA kit was used to prepare cDNA and the library was prepared using the Ion Plus Fragment Library kit (Thermo Fisher Scientific, 4471269).

### Modified MALBAC method

Ribosomal RNA was depleted with the Low Input RiboMinus Eukaryote System v2 kit (Thermo Fisher Scientific, A15027). RiboMinus Magnetic Bead Cleanup (Thermo Fisher Scientific, A15026) was used to concentrate and purify the rRNA-depleted RNA. In the MALBAC method, cDNA was made using primers containing random hexamers and an adaptor sequence: 5’-GTGAGTGATGGTTGAGGTAGCAGTTCAGNNNNNN-3’. cDNA was then linearly amplified using suitable primers for linear amplification as described in the original MALBAC method (16); MALBAC primers also contained the Ion A adaptor sequence and an IonXpress barcode. Final amplification of the library used primers that ligate to the Ion A adaptor and key sequence and to a truncated Ion P1 adaptor. The final library was size-selected using E-Gel SizeSelect Agarose Gels (Thermo Fisher Scientific, G661002).

### Direct ligation method

Total RNA was ribo-depleted with Low Input RiboMinus Eukaryote System v2 (Thermo Fisher Scientific, A15027). RiboMinus Magnetic Bead Cleanup (Thermo Fisher Scientific, A15026) was used to concentrate and purify the rRNA-depleted RNA. Ion Total RNA-Seq Kit v2 was used to make cDNA, add barcodes, and amplify the library. All cleanup steps were performed using Agencourt Ampure XP beads (Thermo Fisher Scientific, A63880).

### Sequencing and whole genome alignment

RNA libraries were sequenced on P1v2 chips using the Ion Proton™ System (Thermo Fisher Scientific, #4476610). Sequencing was completed by the Analytical and Translational Genomics Shared Resource at the University of New Mexico Cancer Center. All samples were aligned with Torrent Mapping Alignment Program (TMAP), using the 1000 genomes GRCh37 phase II d5 the human reference genome. Subsequently, the data were aligned with the STAR aligner [18] using the same reference genome. Exon feature counts were generated with HTSeq-count [19] using a modified RefSeq references, or our own annotations when counting MYB features. Consistent with the Ion Proton sequencing technology, all reads were treated as single-end for the purposes of genome alignment and feature counting.

### Spliced alignments

Cufflinks [20] with the Ensembl 75 reference was used to estimate isoform observations [21]. Subsequent analysis of the data was conducted in R using packages listed in supplemental section 1. RNA sequencing data are available for download from the NCBI Sequence Read Archive using SRA BioProject accession number PRJNA368701.

### Data analysis

The total number of sequenced reads varied by technique and sample. Therefore, to avoid bias towards samples with more total reads, each sample was analyzed as the average of 5 randomly selected subsamples of 10 million reads. The subsampling was done without replacement using a reservoir sample tool [22]. Each time the random subsampling was performed, the same seed value was used for repeatability. The analyses described above were then repeated on the subsamples and results were again tabulated in R using scripts ‘readSummaryFigure’ and ‘subsampleSummaryFigure’ that are available for download [23]. Deviation between the subsamples for a given sample was small and the best subsample for each sample was used to generate the various figures in the paper.

### RNA splicing comparisons

The STAR aligned BAM files for total reads or the 10 million read subsamples were used to calculate the gene and isoform expression count using cufflinks version 2.2.1 [20]. The count information for quantitative expression of unique gene or transcripts was derived by calculating the number of unique splicing sites under the attributes gene_id and transcript_id in the GTF record. The mean and standard error was calculated for the 5 subsamples. The replicate sample data, each containing 10 million read subsamples, were combined to calculate P-value for statistical significance in comparisons between the RP and DL methods for the CD34+ and Jurkat samples.

To compare splice sites that were shared or unique to samples, we combined the chromosome, first-intron base, and last-intron base information from STAR alignment files to make a unique identifier that could be compared across all samples. This script is available as ‘spliceJunctionTable’ (https://github.com/scottness/rna_seq_libraries_analysis). We excluded any splice junction sites from the data analysis that did not contain a read coverage of at least 5 and were not identified in all 5 subsamples. The splice junction sites were then compared across each method to identify the unique and shared junctions that are depicted in Venn diagrams.

### RNA editing comparisons

The RNA editing sites were identified in the study using a modified variant caller format program, ‘editing_site_coverage_example’ (https://github.com/scottness/rna_seq_libraries_analysis). To filter out potential false positives in our data sets, we excluded any sites from our analysis that did not contain at least a minimum of 5 reads in all of the sampling groups for a given method. To exclude known SNP and mutation sites from our data, we filtered against the NCBI dbSNP Build 137 [24] and the COSMIC v72 database respectively [25]. Finally, the sites were filtered to the RADAR [26] and DARNED [27, 28] databases to specifically identify annotated editing sites. The number of editing sites was then tabulated for each sample in total reads and the 5 subsamples containing 10 million reads. The replicate subsample groups were combined to calculate the P-value significance between the RP and DL methods. The unique and shared editing sites between the two methods are depicted in Venn diagrams.

## Results

### Library methods differ in 5’ to 3’ coverage and unique reads

**Fig 1:**
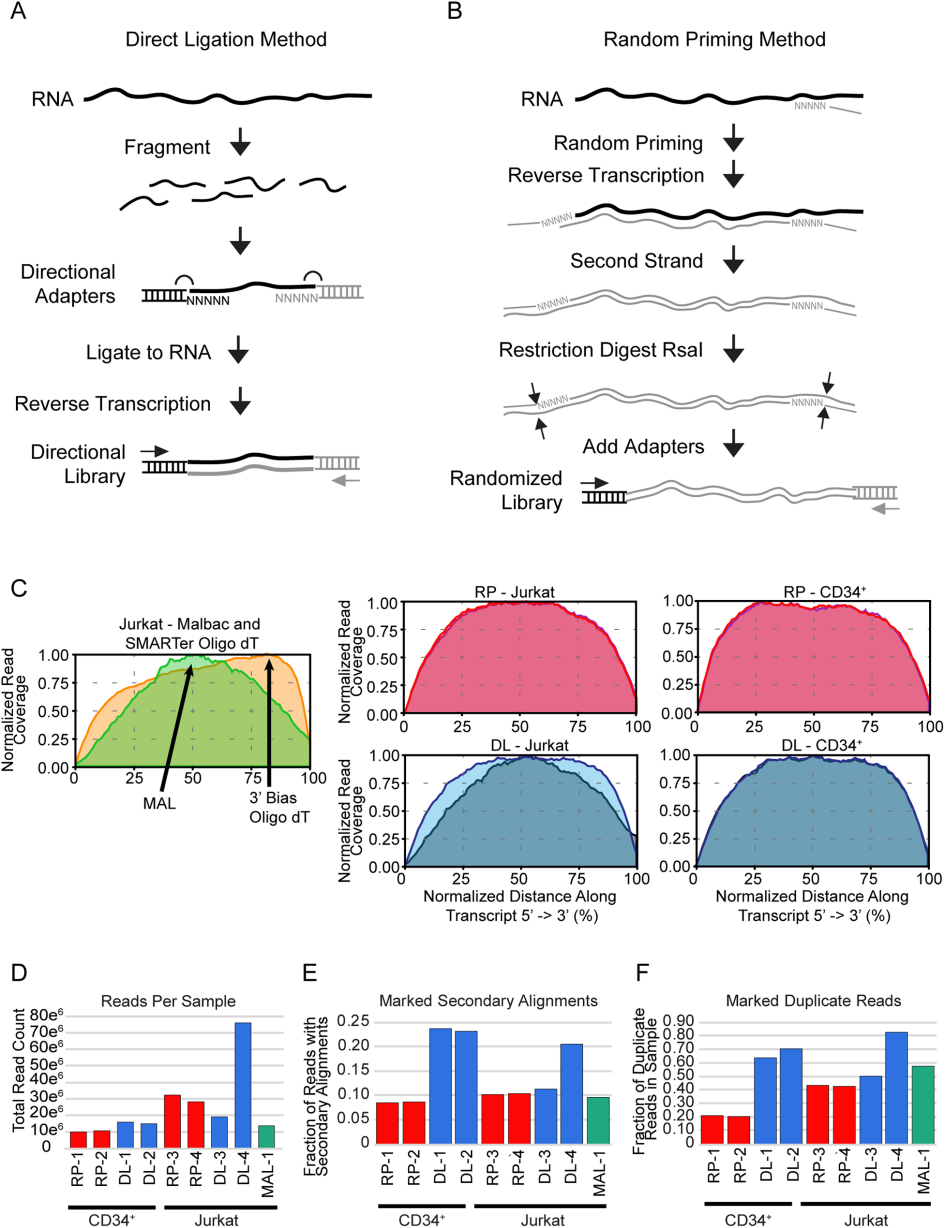
Comparisons of RNA-seq library methods. (A) and (B) Outline of the steps involved in the Direct Ligation (DL) and Random Priming (RP) library methods. Details are provided in Materials and methods. (C) Plots of average read coverage from 5’ to 3’ across the 10,000 most highly expressed genes for each library method. Note the 3’-end bias in the oligo-dT method (yellow) and the extreme center-weighted coverage for the MALBAC method (far left, green). Two independent libraries from the Jurkat (middle) and CD34+ (right) samples were compared for the DL (bottom, blue) and RP (top, red) methods. (D) Reads per sample shows total library size, in reads mapped to the reference genome (larger is better). (E) Marked secondary alignments is an indicator of reads that align to more than one location in the genome (smaller is better). (F) Marked Duplicate Reads is a measure of reads with identical start points and CIGAR scores (smaller is better).

Predicting the structures of proteins encoded by different RNA isoforms produced as a result of alternative RNA splicing requires detailed knowledge of the transcripts and splice junctions as well as even coverage across all the exons included in the transcripts. We tested four common methods of RNA-seq library preparation, using ribosomal RNA-depleted RNA from either human Jurkat T-cell leukemia cells or primary human CD34+ hematopoietic progenitor cells from normal donors. To compare the library methods, we prepared two independent RNA samples from each cell type and prepared independent libraries with each method on different days, using aliquots of the same RNA prep for each set of libraries. The four methods that we tested are described in detail in the Materials and methods section. For each of the commercial methods, we used the ribosomal RNA depletion kits that were recommended and followed the manufacturers recommendations. Briefly, the oligo-dT method used oligo-dT to prime cDNA synthesis from the poly-A tail of mRNAs. The MALBAC method (MAL) used a random 8 nt binding sequence and 27 nt self-complementary common sequence designed to form loops after cDNA synthesis to encourage linear amplification and prevent subsequent exponential amplification that could skew the results. This approach has been described for amplifying and sequencing rare sequences or performing single-cell RNA sequencing [16]. The Direct Ligation (DL, Fig 1A) method relied on random fragmentation of the RNA followed by direct RNA ligation to add adapters, allowing for amplification and cloning and preserving the strand information, which is an advantage for some purposes. The random priming (RP, Fig 1B) method used 6 nt random primers with short common sequences that include an RsaI restriction site to prime double-stranded cDNA synthesis. RsaI digestion removes the adaptors but also results in loss of strand information and cleaves cDNAs that contain RsaI sites, which can affect results.

We first measured the extent of coverage across transcripts by identifying the 10,000 most highly expressed genes, summing the average coverage, and plotting the results (Fig 1C). Both the oligo-dT and MALBAC methods yielded unsatisfactory results, the former because of 3’-end bias and the latter because of inadequate coverage at both ends and over-weighting of the middle of the transcripts (left panel). The random priming (RP) method showed the least bias and most even coverage, both for Jurkat (top, middle) and CD34+ (top, right) samples. The Direct Ligation (DL) method also showed good overall coverage, although weighted more to the center and less consistent than the RP approach, both for Jurkat (bottom, middle) and CD34+ (bottom, right). Based on these results we focused only on the DL and RP methods for the rest of our analyses, although we have included some results obtained with the MALBAC method for comparison.

### Secondary alignments and duplicate reads provide insight into library quality

To gain a deeper understanding of the differences between the library methods, we analyzed features of the RP and DL results in detail. We compared the results from 6 different sequencing runs on the Ion Proton platform: CD34+ cell RNA with libraries prepared with either the RP or DL methods, all of which yielded 10–20 million mapped reads per library, or Jurkat T-cell line RNA prepared similarly, which yielded 20–30 million mapped reads per library (Fig 1D). The exception was the Jurkat library DL-4, where a single library was analyzed on an entire P1 sequencing chip to test whether more total reads overall would help with the splicing analysis (discussed below). We have also included the results from MALBAC Jurkat T-cell library, which also had more than 10 million mapped reads.

We first analyzed the fraction of reads from each library marked as secondary alignments, indicating that they aligned with more than one position in the genome (Fig 1E). Although the RP and MALBAC libraries yielded fewer than 10% reads marked as secondary, three of the four DL libraries resulted in 20% or more reads with secondary alignments. These are likely to be low-complexity or repeated sequences. An analysis subsequently showed that the DL libraries had a large number of reads mapped to repetitive snoRNA sequences, which reflect a difference in the ribosomal depletion methods that were used.

We also plotted the fraction of duplicate reads, defined as having identical start positions and CIGAR strings (Fig 1F). The RP libraries gave the lowest fractions of duplicate reads. Interestingly, the DL method libraries yielded more than 50% duplicate reads in our tests. This suggests that the standard conditions used in the DL method result in over-amplification and a higher fraction of reads that are duplicates.

These two quality control metrics, secondary reads and duplicate reads, showed important differences between the ribosomal RNA depletion kits and the amplification approaches that have little impact on gene expression measurements, but may have important implications for assessing more advanced transcriptome features, such as alternative RNA splicing.

### Differences in detection of genes and isoforms

**Fig 2:**
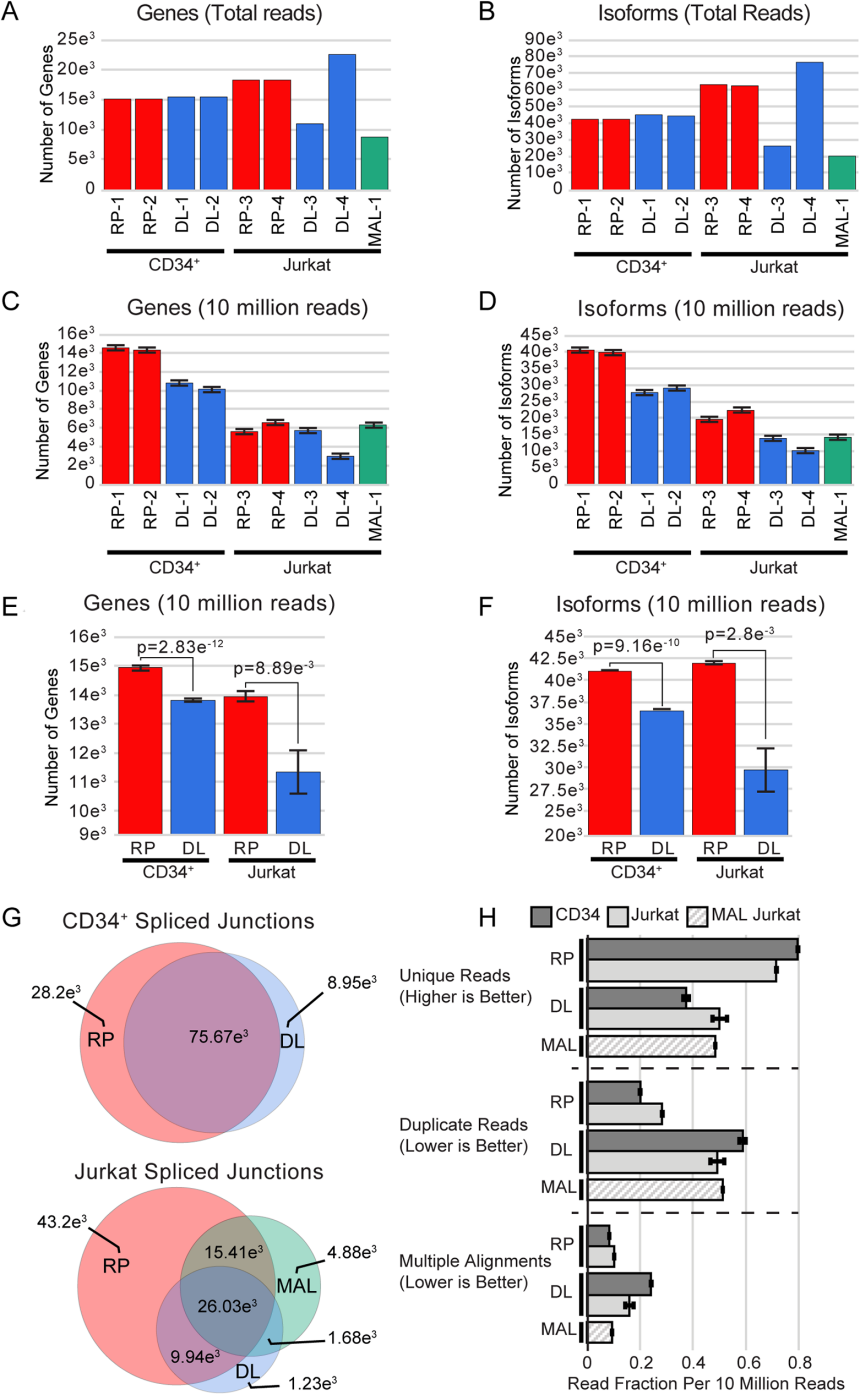
Genes and isoforms comparisons. Cufflinks analysis was performed for (A) Gene number (B) and isoform number for each cell line and method, using total read count data. A similar analysis using (C) mean gene number and (D) mean isoform number obtained from 5 independent sampling events of 10 million reads from each sample. The 5 independent random samples of 10 million reads from each of the replicate samples were combined to give 10 total independent random sample events from replicates for testing the significance in a t-test for (E) gene number and (F) isoform number. (G) Venn Diagrams of STAR junction reads from CD34+ (top) and Jurkat (bottom) libraries, highlighting the differences in junction read coverage with each method. (H) Graph summarizing the three methods, following normalization of total alignments from the 10 million read count samples, including unique reads, duplicate reads, and secondary alignments.

To test the usefulness of the libraries for transcriptome analyses, we analyzed the total number of genes that were detected (as expressed above a base threshold) and the total number of different splicing variant isoforms detected in each library. Using the total data set from each sequencing run showed that both the RP and DL methods detected approximately 15,000 expressed genes in the CD34+ sample. As shown in (Fig 2A), both of the RP libraries detected approximately 18,000 (RP) expressed genes in the Jurkat sample. We were surprised to find that the DL-3 library only detected about 11,000 expressed genes in the Jurkat sample, approximately 40% less than the RP libraries. This is likely due to the relatively high number of secondary alignments (snoRNA) in the DL libraries. Therefore, we ran the second library (DL-4) by itself on a P1 sequencing chip, generating nearly 80 million reads, and that sample detected more than 22,000 expressed genes. Thus, using 4 times more reads led to the detection of about twice as many expressed genes in the Jurkat cells.

We observed very similar results when counting the number of different splice variant isoforms detected in each library. Both the RP and DL methods detected over 40,000 transcript isoforms in the CD34+ samples. The RP method detected over 60,000 isoforms in the Jurkat samples. The first DL library, DL-3, detected only 27,000 isoforms in Jurkat, but using 4 times more reads (DL-4) led to the detection of nearly 80,000 transcript isoforms in Jurkat cells. Thus, increasing the number of reads increases the sensitivity of the transcriptome assays and the number of expressed genes or isoforms detected. However, using more reads also increases the cost of the RNA-seq assays.

As shown in Fig 1D, the numbers of reads in these libraries differed substantially, and the costs of performing RNA-seq experiments are linked closely to the number of reads obtained. Therefore, we also calculated the genes and isoforms detected when a constant number of reads were used for each library method. As shown in (Fig 2, panels C and D), the results changed dramatically when a total of 10 million reads was randomly sampled from each data set. For the CD34+ samples the RP and DL methods detected approximately 14,000 and 11,000 genes, respectively, and 40,000 and 29,000 isoforms, respectively. For the Jurkat samples, the RP libraries detected 6,000 genes and 21,000 isoforms. Although the first DL library detected 6,000 genes, only 15,000 isoforms were detected, nearly 30% less than with the RP method. When only 10 million of the 80 million reads from the DL-4 library were analyzed, only 3,000 genes and 11,000 isoforms were detected. This poor result is likely due to the high number of secondary alignments and duplicate reads that occurred when the single DL-4 library was run by itself on an entire P1 chip (Fig 1, panels E and F).

To check for sampling errors, we randomly sampled each library 5 times, generating independent 10 million read subsamples, then averaged the gene and isoform detection results. The results, shown in (Fig 2E and 2F), show that the RP method performed best at both gene and isoform detection, when equal numbers of reads were compared. Because of the high number of snoRNA reads, and the higher number of duplicate reads, the DL method led to the detection of significantly fewer genes and isoforms for both the CD34+ and Jurkat samples. To further validate that the RP method identified more data points in splicing, we calculated the number of STAR-aligned unique splice junction sites that appeared in all 5 subsample groups and both of our replicate samples. In CD34+ samples (Fig 2G), we identified 28,200 unique junctions for the RP method, compared to only 8,950 unique junctions for the DL method. In the Jurkat samples, that included RP, DL, and MAL for comparison, these differences were even more significant with the RP method having 43,200 unique splice junction sites as compared to only 4,880 junctions in the MAL and 1,230 junctions unique in the DL methods. In total 102,370 splice junctions were identified in Jurkat cells, 94,580 or 92.4% of these were identified using the RP method. In the MAL samples, 48,000 or 46.9% of the unique junctions were identified, and the DL method only identified 38,880 or 38% of the unique junctions.

The results of these assays are summarized in (Fig 2H), by showing the secondary alignments, duplicate reads, and unique reads detected by each method, averaged across the 10 million read subsamples and normalize to put all the measures on the same scale. The RP method performed best in all of the metrics.

### RNA-editing comparisons

**Fig 3:**
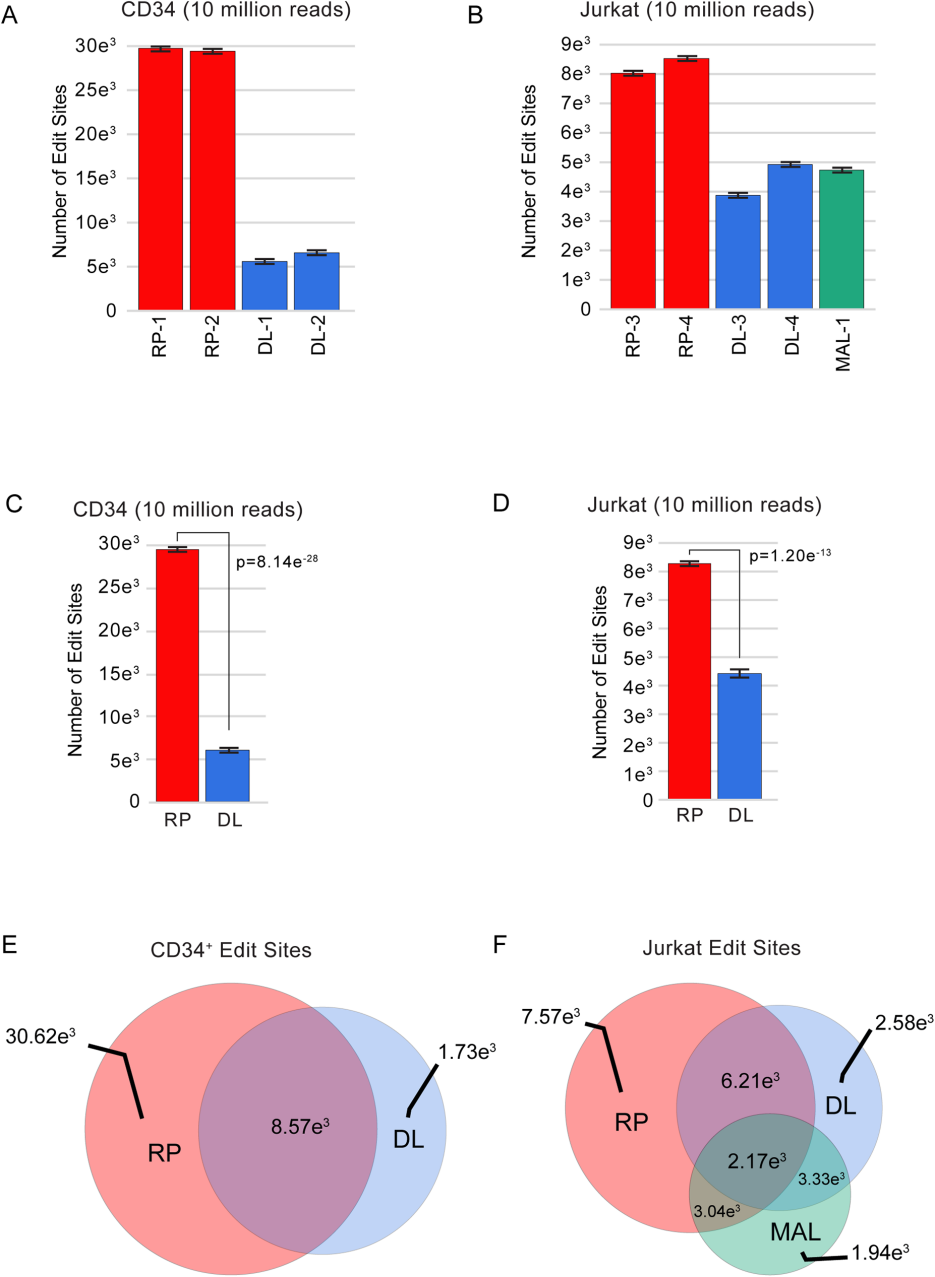
Number of RNA editing sites. (A) CD34+ cells and (B) Jurkat cells. Replicate samples were combined to give 10 total independent random sample events from replicates of (C) CD34+ and (D) Jurkat cells for testing the significance in a students’ t-test. Venn Diagram of Random Primer editing site overlaps for (E) CD34+ and (F) Jurkat cells.

RNA editing results from post-transcriptional enzymatic modifications lead to apparent changes in the RNA sequence. Changes in RNA editing have recently been implicated as important for cell differentiation and in cancer [1, 29]. We analyzed our RNA-seq data for potential RNA editing events, using only the detected variants that were previously listed in on-line databases of known RNA editing sites [30, 31]. As shown in Fig 3A, in the CD34+ samples, the DL method had three times the number of duplicate reads as the RP method, resulting in the detection of six fold fewer RNA editing sites. In the Jurkat samples (Fig 3B), the RP method identified >2 fold more editing sites, even when sharing a comparable number of duplicate reads. This two fold higher number of RNA editing events detected in the RP libraries cannot be directly explained by the number of duplicate reads and is likely the result of differences in coverage across the genome. The differences were statistically significant, when averaged across multiple 10 million read subsamples (Fig 3C and 3D).

We also tested whether the methods detected the same or different sets of RNA editing sites. As shown in the Venn Diagrams in Fig 3E and 3F, most of the RNA editing sites detected by the DL method were also present in the larger RP method groups. However, there were some putative editing events that were detected uniquely by each method, suggesting that the methods provided different types of coverage over the transcriptome.

### Focusing on MYB splice variants reveals important differences

**Fig 4:**
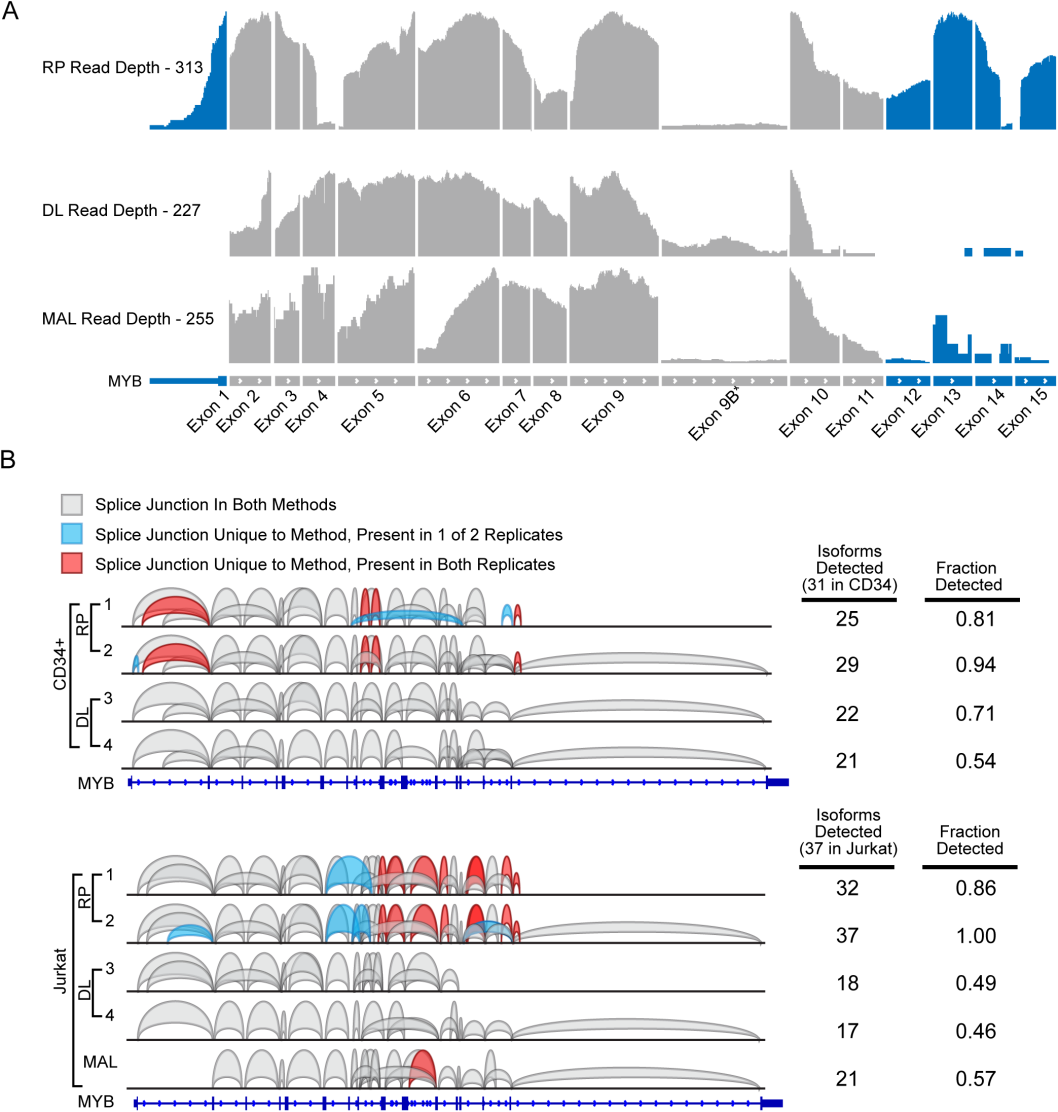
Detection of unique isoforms. (A) Read distribution across the MYB gene for DL (top), RP (middle), and MAL methods (bottom). (B) Sashimi Plot of MYB splice junctions identified using the RP or DL method in CD34+ cells (top) or Jurkat cells (bottom). Junctions shaded blue or red were exclusive to a single method.

The human MYB gene can produce as many as 60 different RNA isoforms through alternative RNA splicing, which encode at least 20 different versions of the Myb protein [7, 8]. We compared the coverage and splice variants detected in the MYB gene as a final measure of the advantages and disadvantages of the different library methods. Fig 4A shows the coverage plots for 10 million read subsamples for the RP, DL, and MALBAC methods, with coverage aligned to the 15 most common MYB exons. The RP method (top) was the only one that reliably detected all of the exons, including the first and last exons and untranslated regions. (Note that coverage of exon 9B, sometimes labeled exon 10, is much lower, as it is an alternatively spliced exon that is rarely used in these cells.) The RP method also failed to adequately capture exon 4. This is due to the RsaI restriction digest used in the preparation of the RP libraries. MYB exon 4 contains a naturally occurring RsaI site, which affects the detection of that exon by the RP method. The DL method gave fairly even coverage of MYB exons 2–10, but failed to capture the ends of the transcripts. That was also true for the MALBAC method, which failed to capture the 5’ and 3’-ends of the MYB transcripts.

Finally, we analyzed the numbers and locations of MYB transcript isoforms detected by each RNA-seq library method. We started with 10 million reads selected from each data set and filtered the RNA-seq results to limit our analysis only to junction reads, identified as spanning two or more exons, and plotted the results in the “Sashimi Plots” shown in Fig 4B. In these plots, the splice events are represented as curves connecting two exons. The splice junctions common to all the libraries are shown in gray. The ones that were unique to a single method are shaded blue or red for emphasis. The numbers at the right show the number of MYB isoforms detected in each library and the fraction of the total isoforms detected by each library. The top panel shows the results for the CD34+ sample: all of the junctions detected in the DL libraries were also detected in at least one of the RP libraries. But the latter also revealed several unique junctions that were not detected in the DL libraries. Similar results were found for the Jurkat samples, in the lower panel. Thus, the RP method of library preparation appears to be superior to the DL method for detection of alternative splicing isoforms, either when sampling across the entire genome (Fig 2) or when analyzing a single gene such as MYB.

## Discussion

We tested several RNA-seq library preparation methods, using both popular commercial kits and homemade approaches, to find the advantages and disadvantages of each and to determine how best to analyze advanced features of the transcriptome, such as RNA editing and alternative RNA splicing. One of the most important findings was a strong correlation between the total number of reads analyzed and the sensitivity of the assays, measured in terms of expressed genes or isoforms detected. This relationship is well known and is reflected in the ENCODE Consortium best practices guidelines [32], which advocate the use of at least 30 million reads for detecting rare transcripts or isoforms, but significantly less for comparing transcription profiles. We chose a read depth of 10 million mapped reads for our base comparisons, which is routinely achievable when analyzing four independent libraries on a single Ion Proton P1 chip. We found marked differences in the results, due in part to differences in the ribosomal depletion kits that were recommended by different manufacturers, and to the differences in the RNA-seq library methods.

We found a major difference in the results obtained when using different ribosomal RNA depletion kits. The kit recommended for use with the DL approach did not remove the very abundant snoRNAs, and those sequences consumed a relatively large fraction of the resulting RNA-seq libraries. For researchers focused primarily on protein-coding genes, the use of a different ribosomal RNA depletion method, such as the kit we used in the RP method, would be a better approach. However, this demonstrates how choices that seem relatively innocuous, such as the choice of ribosomal RNA depletion kit, can have a major impact. On the other hand, data sets generated using kits that remove snoRNAs cannot be used for some purposes where analysis of snoRNAs is important.

Another difference in the methods is whether they preserve the strand information of the transcripts. For many purposes, such as gene expression measurements, preservation of strand information may not be important, but it is critical for measuring transcripts from overlapping genes. The RP method we used was better at most metrics that we analyzed but did not conserve the strand information, which is a drawback. The DL method did preserve the strand information for transcript reads but had other disadvantages. These issues should be considered carefully by investigators before designing an RNA-seq approach.

Our analysis of MYB gene alternative splicing detected at least 29 different transcript isoforms in both CD34+ hematopoietic progenitors and in Jurkat T-cells, which is consistent with previous reports [7, 8]. However, we found that the choice of RNA-seq library method was critical for detection of the most isoforms. When balanced for the same number of mapped reads, the RP method detected at least 25% more transcript variants than the DL or MALBAC methods. The RP method was also superior for detecting RNA editing events. These results illustrate the importance of designing RNA-seq experiments using the optimum library preparation methods and collecting sufficient reads to be able to adequately measure advanced transcriptome features such as alternative RNA splicing and RNA editing, and also point out possible issues when comparing data sets that were produced using different methodologies.
